# Assessing the gut microbiota composition in older adults: connections to physical activity and healthy ageing

**DOI:** 10.1007/s11357-025-01605-w

**Published:** 2025-03-17

**Authors:** Catarina Ramos, Daniele Magistro, Gemma E. Walton, Anya Whitham, Nicola Camp, Carlos Poveda, Glenn R. Gibson, John Hough, Will Kinnear, Kirsty Hunter

**Affiliations:** 1 Department of Sport Science, Sport, Health and Performance Enhancement (SHAPE) Research Centre, Nottingham Trent University, Nottingham, UK; 2 Department of Food and Nutritional Sciences, The University of Reading, Whiteknights, Reading, UK; 3 Reynolds Contamination Control, Lincoln, UK

**Keywords:** Gut microbiota, Older adults, Ageing, Gut microbiome, Physical activity, Exercise, Elderly, Healthy ageing

## Abstract

The composition and functionality of the gut microbiota (GM) changes throughout the life course. As we move into older age, it starts to shift towards a less healthy one, which may lead to an imbalance in the GM community. Strategies that can reverse age-related dysbiosis are an important part of healthy aging. Little is known about the GM composition of older adults with different physical activity (PA) levels and whether it might contribute to healthy ageing. The aim of this study was to compare the GM composition of older adults with different PA levels and assess if it is associated with healthy ageing. 101 participants aged between 65–85 years undertook anthropometric measures, a 6-min walking test, wore an accelerometer for 7 days and provided a faecal sample. Faecal GM composition was analysed using 16S rRNA sequencing. We found that those who fulfilled the WHO/UK PA recommendations had higher relative abundance of several health-related bacteria such as *Lactobacillus, F. prausnitzii* and *Roseburia intestinalis* and lower abundance of disease-associated bacteria such as *D.piger* or Enterobacterales when compared to those who did not reach PA recommendations. These findings suggest that PA might improve the GM composition and has the potential to, at least partially, revert age-associated dysbiosis and promote healthy ageing.

**Supplementary Information:**

The online version contains supplementary material available at 10.1007/s11357-025-01605-w.

## Introduction

In 2022, there were 771 million people aged over 65 years worldwide, and it is expected that the number of people in this category will reach 994 million by 2030 and 1.6 billion by 2050 [1]. Additionally, it is projected that by 2050, the number of people aged above 65 years will be more than twice the number of children aged below 5 years and about the same as the number of children aged below 12 years [1].

The global population is not only getting older but also more inactive; around 1 in 4 adults do not reach the recommended physical activity (PA) levels [2]. Older adults spend, on average, 65–80% of their waking time sitting [3]. This is concerning, as physical inactivity is responsible for more than 7% of all-cause mortality and up to 8% of non-communicable diseases [4]. Thus, PA is an important promotor of healthy ageing, which is defined as the process of developing and maintaining functional ability that enables wellbeing in older age, including the person’s ability to meet their basic needs; be mobile; make their own decisions; build and maintain relationships and contribute to society [5].

A mechanism by which PA might improve overall health and promote healthy ageing in the older population is via the gut microbiome. The gut microbiome is the totality of the mixed community of gut microorganisms, including genetic components and their resulting functionality [6]. These microbes can impact the host by influencing numerous physiological systems and whole-body metabolism through multiple mechanisms including the release of bioactive metabolites which impact on body cells locally and systemically. For example, short-chain fatty acids (SCFAs) can regulate glucose and cholesterol metabolism [7] and modulate the immune system [8]; and the Gram negative bacterial cell wall component lipopolysaccharide (LPS) has been associated with insulin resistance [9], gut permeability and inflammation [10]. Based on the above, it is easy to understand that any alteration of the composition and functionality of the gut microbiome influences the whole body and that these alterations may be involved in the pathophysiology of both acute and chronic diseases.

The gut microbiome composition and thus, its activity, varies during different life stages; during adulthood it tends to remain fairly stable, however, with the transition into older adulthood it changes towards a more unbalanced state which normally consists of an increase in pathobionts, a decrease in health related bacteria, a decrease in diversity and large interindividual variability, such a change is sometimes referred to as dysbiosis [11, 12] The compositional shift coincides with the onset of immune dysregulation and manifestation of ageing-associated pathologies, these can include stroke, Alzheimer’s disease and cancer. Strategies for healthy ageing should ideally slow the rate of dysbiosis, and thus, its contribution to ill health.

There are several factors that may contribute to these microbial changes in older age, such as consumption of a more monotonous diet [13], reduced appetite and thus reduced nutrient and energy intake (a phenomena called “anorexia of ageing”) [14], alterations in gut physiology (e.g. reduced intestinal motility) [15], antibiotic treatments [16–18] consumption of NSAIDs [19], polypharmacy [20], a weakened immune system [21] and living arrangements [22]. The resulting gut microbial changes might then lead to increases in gut barrier permeability which can enhance the leakage of bacterial components (e.g. LPS) or metabolites to systemic circulation, consequently activating inflammation cascades. This, combined with the immunosenescence that occurs during the ageing process, will increase the inflammatory state and can contribute to several diseases/conditions, such as obesity [23], type 1 diabetes [24], type 2 diabetes [25], inflammatory bowel diseases (IBD) [26], frailty [12], insulin resistance [27], cardiovascular diseases (CVDs) [28, 29], asthma [30], colorectal cancer [31], stress- related disorders [32], dementia [33], hypertension [34], Alzheimer’s disease [35], Parkinson’s disease [36] and rheumatoid arthritis [37]. Furthermore, perturbations in this tightly regulated ecosystem will affect energy metabolism, nutrient absorption, appetite regulation, the immune system and the synthesis of several key metabolites, such as SCFAs and vitamins. Thus, interventions to change the gut microbiome to a ‘younger’, less dysbiotic profile are desirable in older adults as part of a successful and healthy ageing process.

Although still a relatively new area of research, several studies have shown that PA and exercise are associated with an altered gut microbiome both in terms of microbial composition and functional activity. Barton and colleagues [38], for example, compared the gut microbiota composition of professional rugby athletes with sedentary controls and demonstrated that their gut microbiotas were distinctly different. Athletes had higher microbial diversity and more SCFA producing bacteria when compared to the sedentary controls. The same conclusion in relation to PA level was reached by Bressa and colleagues [39], who found that pre-menopausal women who were active had a different microbiota composition when compared to women who were sedentary. More specifically, healthy women had a higher abundance of health-associated bacteria, such as *Faecalibacterium prausnitzi*, *Roseburia hominis* and *Akkermansia muciniphila*. Moreover, they also found an inverse association between microbial diversity and sedentary behaviour and a correlation between body fat, age, muscle mass and PA with several bacterial populations. These previous studies demonstrate that PA is associated with a health-associated gut microbiota, however, none of these studies controlled for the influence of diet as a confounding factor. It is possible that people who are physically active tend to have a healthier diet when compared to inactive or sedentary people, and this can affect the results. Allen and colleagues [40], however, demonstrated that 6 weeks of endurance exercise training was able to induce changes in the gut microbiota in lean but not in obese participants independently of diet. In this study exercise was shown to increase the concentration of faecal SCFAs and the bacteria that produce them. However, the exercise-induced changes in the gut microbiota went back to baseline after a 6-week washout period that required the participants to return to their sedentary lifestyle. These findings suggest that continuation of exercise may be needed in order to maintain or improve the changes that occur in the gut microbiota, as is the case with dietary modification.

Despite these initial reports in younger adult populations, little is known if older people who are more active have a different gut microbiota composition compared to those who are not. Moreover, knowledge on whether exercise or PA interventions can positively influence the gut microbiome of older adults, healthy or otherwise, is limited with only one intervention study from Japan [41], one from China [42], and one from the USA [43] and four cross-sectional studies [44–47] performed, to our knowledge. A systematic review performed by our research group [48] concluded that PA is beneficial to improve the GM of older adults, however, more clarification is needed to understand which taxa are more sensitive to PA and to understand the specific changes induced by PA/exercise on the GM of the ageing population. Therefore, the aim of this study is to compare the gut microbiota composition of older adults with different PA and cardiorespiratory fitness levels.

## Methods

### Study design and participants

This was an observational, cross-sectional study that assessed the gut microbiota composition and PA levels of 101 non-smoking, healthy, community dwelling older adults aged between 65 to 85 years from the UK. A total of 131 older adults were recruited to take part in the study; 30 were excluded because they did not fulfil the inclusion criteria.

Participants were given the option to either visit the laboratory once (one visit) or twice (two shorter visits) depending on their personal schedule. All visits started at 9am. Participants were asked to refrain from consuming caffeine or alcohol during the 24 h before the study visit and to refrain from engaging in strenuous PA on the day before their visit. Additionally, they were asked to refrain from eating for at least 10 h before visiting the laboratory.

#### Inclusion and exclusion criteria

The inclusion criteria were: (1) aged between 65–85 years old; (2) community-dwelling; (3) fully vaccinated against COVID-19 and (4) with a BMI between 18.5–35.0 kg/m^2^. The exclusion criteria were: (1) routine consumption of nonsteroidal anti-inflammatory pharmaceuticals (NSAIDs); (2) consumption of antibiotics 3 months before or during the study; (3) cancer; (4) chronic kidney disease; (5) intestinal inflammatory conditions – inflammatory bowel disease (including ulcerative colitis and Crohn’s disease); (6) auto-immune diseases; (7) gastrointestinal diseases; (8) smoking and (9) routine consumption of pre and/or probiotic foods or supplements.

### Health questionnaire

Participants completed a thorough health questionnaire to determine their clinical history and overall health and to check if they were suitable to take part in the study. A medical advisor was responsible for checking the health questionnaires and either including or excluding the participants from taking part in the study.

### Assessment of physical activity

The level of PA was assessed over 7 days using the tri-axial accelerometer Actigraph wGT3X-BT (Actigraph, Pensacola, Florida, USA) placed on the waist. The assessment period of 1 week was to allow for variations in PA during weekdays and the weekend. Level of PA was then calculated using the actigraphy data analysis platform Actilife Software (version 6.13.4, Actigraph). The accelerometer included an inclinometer which allowed the assessment of time spent sitting, lying down and standing up.

Participants were advised that the device was not waterproof and therefore to remove it before engaging in any water-based activities. These participants were asked to provide an exercise diary that stated which water-based activity they participated in, for how long and any other information that they might have such as meters swam, for example. Additionally, some participants took part in cycling activities and if the accelerometer was placed on the waist, these might not have recorded properly, therefore participants were also asked to provide an exercise diary stating the time spent and distance cycled. The time spent exercising for the above activities was then included in the calculation of PA.

Participants were also told that if the device caused any discomfort during sleep that they could take it off during that period since, in this study, daytime activity level was the only variable of interest.

In terms of data processing and analysis, the device was set up to collect units of gravity (1 g = 9.82 m/s^2^) at a sample rate of 100 Hz per second. Data were downloaded and processed using the normal filter option into 30 s epochs. The Choi’s [49] algorithm was used to calculate the wear-time validation (non-wear time was defined as 90 consecutive minutes of 0 counts) and included the following optional parameters: Minimum wear time p/day: 420 min; Minimum weekdays wear time: 3; Minimum weekend days wear time: 1. All non-wear times were excluded from the analysis. Counts per minute (CPM) were used to define PA intensities, and the Freedson adult VM3 (2011)’s cut points are as follows [50]: light: 0–2690 CPM; moderate: 2691–6166 CPM; vigorous: 6167–9642 CPM; very vigorous: > 9643 CPM. Sedentary behaviour was defined as < 100 CPM.

#### Criteria for classifying participants based on their PA levels and number of steps/day

Moderate to vigorous PA (MVPA) was used to classify the PA level of the participants. Briefly, MVPA was categorised into five levels based on multiples of the minimum PA recommendations by the WHO and the UK Chief Medical Officers, as shown below in Table 1. Additionally, the number of steps/day was grouped according to average number of steps the participants did per day.

**Table 1:** Criteria for the definition of the PA levels and the number of steps/day

Category	Group	Definition of the category
MVPA/week	Inactive	< 150 min/week (below PA recommendations)
	150–300 min/week	150–300 min/week (1–2 × PA recommendations)
	300–450 min/week	300–450 min/week (2–3 × PA recommendations)
	450–600 min/week	450–600 min/week (3–4 × PA recommendations)
	More than 600 min/week	> 600 min/week (> 4 × PA recommendations)
Steps/day	Less than 6000 steps/day	Did less than 6000 steps/day
	Between 6000–9000 steps/day	Did between 6000–9000 steps/day
	Above 9000 steps/day	Did above 9000 steps/day

### Cardiorespiratory fitness test

The 6-min walking test (6MWT) is a sub-maximal test used to assess cardiorespiratory fitness. BP, heart rate and the rate of perceived exertion (RPE) [51] were taken at the end of the test to evaluate the cardiovascular system’s response to the exercise. RPE is a subjective method to assess exercise intensity. It consists of a scale from 6–20 that reflects the individual’s perception of physical effort after the exercise; the number 6 represents the resting state, and the number 20 represents maximal exertion. Briefly, older adults were asked to assign a number on the scale that best reflected how hard they felt they were working. These data along with BP and heart rate were used in a previously validated equation [52] to predict maximal oxygen uptake (VO_2_max) based on the 6MWT.

#### Criteria for defining the groups for the 6MWT

The distance walked in the 6MWT was used as a proxy for cardiorespiratory fitness and participants were divided into quartiles (Table 2) based on the distance they were able to cover during the test.

**Table 2:** Criteria for the definition of the 6MWT groups

Category	Group	Definition of the category
Distance walked in the 6MWT	Walked less than 472 m	First quartile
	Walked between 472–531 m	Second quartile
	Walked between 531–594 m	Third quartile
	Walked more than 594 m	Fourth quartile

### Assessment of food intake

Estimation of nutrient intake was undertaken using a self-administered food frequency questionnaire (FFQ) validated for the UK population—EPIC-Norfolk FFQ [53]. Participants were asked to recall their dietary intake during the last 6 months. FFQ data was processed using the open-source FETA software (version 6 (CAMB/PQ/6/1205) of the EPIC-Norfolk FFQ) [54] to estimate the amounts of macronutrients, minerals, vitamins and fibre present in their diet.

### Blood sampling and analysis

Fingerprick blood samples were collected following an overnight fast (> 10 h) using a capillary collection tube (Microvette® CB 300, Sarstedt, Germany) coated with lithium heparin. The samples were immediately centrifuged at 4000 g for 4 min at 4 °C and the plasma was extracted, aliquoted and stored at −80 °C until analysis. On average 180 µL of plasma was harvested per participant. The analysis of the biomarkers was carried out using an automated clinical chemistry analyser – Horiba Pentra C400 (Horiba ABX). The intra- and inter-assay coefficients of variation (CVs [%]) for concentrations of HDL cholesterol were 2.05 and 3.27 respectively, for triglycerides were 2.42 and 0.72 respectively, for glucose were 0.61 and 5.61 respectively and for total cholesterol were 0.61 and 3.47 respectively.

### Resting heart rate and blood pressure

Participants were asked to remove any tight clothing or long-sleeved shirts and to sit comfortably for 5 min on a chair with their feet on the floor, legs uncrossed and with their forearms resting on the table or a cushion at the level of their heart. BP was measured twice using an automatic blood pressure device (OMRON, Brighton, UK). If there was a difference greater than 10 mmHg between the first and the second measurement, an additional measurement was taken in order to ensure the most accurate values were obtained. BP was obtained by calculating the average between the measurements. Resting heart rate (HR) was derived from the blood pressure device and the lowest HR value was used.

### Anthropometric measures

All anthropometric measures were performed in the morning and in a fasted state.

Height, weight, waist and hip circumference were measured, and body mass index (BMI) and waist-to hip ratio were derived from these. Before taking the anthropometric measurements, participants were asked to remove any footwear or headwear or heavy/thick clothing, as well as empty their pockets. Height was measured using a stadiometer and participants were instructed to look straight ahead in the Frankfort horizontal plain and take a large breath in. For waist circumference, the measurement was taken either over the skin or, if the participant was not comfortable removing clothing, over light clothing. Waist circumference was measured in the narrowest part of torso. Hip circumference was measured over the widest part of the buttocks. Each one of these measures were performed three times and averaged.

### Faecal sample collection and gut microbial DNA extraction

Faecal samples were collected by participants using a commercial 1 L container (EZ1 Pots with lids, EW Gregory Ltd, UK), aliquoted in triplicates and stored at −80 °C within 2 h of production.

Microbial DNA was isolated and extracted using the QIAamp PowerFecal Pro DNA Kit (QIAGEN, Hilden, Germany) from 250 mg of faeces. DNA extraction was then performed according to manufacturer’s instructions. The end product was 20 µL of purified DNA with a concentration ranging from 10–50 ng/µL per sample. Extracted DNA was stored at −20 °C until sequencing.

### 16 s rRNA sequencing and OTU assignment

Faecal DNA samples were shipped to an external company (Eurofins Genomics, Constance, Germany) and sequenced using Next Generation Sequencing. The V3-V4 (fwd: TACGGGAGGCAGCAG and rev: CCAGGGTATCTAATC) hypervariable regions of the 16sRNA were amplified using Illumina miSeq (PE300 mode). The raw sequencing data was processed using Cutadapt software [55] which finds and removes adapter sequences, primers, poly-A tails and other types of unwanted sequences. The sequences were then subject to quality control where they were demultiplexed, subject to primer clipping, merged and filtered. All reads with ambiguous bases were removed and the chimeric reads were identified and removed based on the UCHIME algorithm [56]. Chimeras are artifact sequences formed by two or more biological sequences that were incorrectly joined together. The high-quality reads were then processed using the minimum entropy decomposition method which is an efficient OTU-picking strategy that can identify and filter random noise in the dataset and posit, allows decomposition of sequence data sets with a single nucleotide resolution outperforming the classical identity-based clustering algorithms. OTU assignment was performed using the QIIME software package and the abundances of bacterial taxonomic units were normalized using lineage-specific copy numbers of the relevant marker genes to improve estimates.

### Statistical analysis

The statistical analysis was performed using R and the Microbiome Analyst 2.0 platform [57]. The biom file containing sample, sequencing data and taxa information was used as a starting point for the analysis in both software.

#### Alpha and beta diversity analysis

The relative abundance and beta alpha diversity indices were calculated/created in the Microbiome Analyst platform.

Alpha diversity is the measurement of diversity within a single community. It is normally categorized using the total number of species (richness), the relative abundances of species (evenness) or indices that combine both of these. Beta diversity is the measurement of diversity between two more communities and provides a measure of the degree to which the samples differ from one another.

Alpha diversity indices (Chao1, ACE, Shannon, observed features, and Simpson) and beta diversity indices (Bray–curtis, Jaccard’s index, Jensen-Shannon divergence) using both Principal Coordinate Analysis (PCoA) and non-metric Multidimensional Scaling (NDMS) as ordination methods were calculated using the MicrobiomeAnalyst 2.0 platform. The significance of the beta diversity indices between the different groups was analysed using a permutational multivariate analysis of variance (PERMANOVA) that was corrected for multiple testing using False discovery rate (FDR) correction. The significance between alpha diversity indices between the different groups was analysed using T-test/ANOVA. The p-value cut-off for alpha and beta diversity analysis was ≤ 0.05.

#### Differential abundance analysis

Differential abundant taxa across the different groups were identified at six taxonomic (phylum, class, order, family, genus, species) using the ANCOMBC2 (version 2.4.0) [58] package in R and RStudio (version: 2023.09.1 + 494). ANCOMBC2 was executed with a prevalence cut-off of 0.1, and statistical significance was defined as alpha ≤ 0.05. Dietary components (carbohydrate, fat and protein intake) and age were used as covariates in all the analyses to account for potential confounding effects.

To adjust for multiple testing and conceptualising the rate of type I errors, the False Discovery Rate (FDR) correction using the Benjamin-Hochberg [59] procedure was applied and the statistical significance was accepted if the FDR corrected P-value (q-value) was ≤ 0.05.

The results for the differential abundance analysis are reported as log-fold changes, a measure used to express a relative change between two variables.

## Results

### Participant characteristics

The study population consisted of 101 community dwelling older adults (63 females and 38 males) aged between 65–85 years and living in the UK. Their main characteristics are summarized in Tables 3 and 4. Their macronutrient consumption as a percentage of energy intake follows the SACN statement on nutrition and older adults living in the community [60], suggesting their diet is generally representative of the UK population.

**Table 3:** Age, sex, medications and anthropometric measures of the participants included

Characteristics		Total *n* = 101 (presented as means ± SD)
Age (years)		71.9 ± 5.3
Sex	Females, n (% of sample)	63 (62%)
	Males, n (% of sample)	38 (38%)
On medication n (% of total)	Epilepsy	4 (4%)
	BP	28 (28%)
	Asthma	6 (6%)
	Metformin	1 (1%)
	Statins	21 (21%)
	Anti-depressants	12 (12%)
	Medication for heart disease	10 (10%)
	Anticoagulants	3 (3%)
	Thyroid	7 (7%)
	Antihistamines	1 (1%)
	Oestrogen	2 (2%)
	Incontinency	2 (2%)
	Eye diseases (macular degeneration; ocular pressure, glaucoma)	3 (3%)
	Osteoporosis	1 (1%)
	Prostate	4 (4%)
Anthropometric measures	Height (cm)	167.1 ± 7.7
	Weight (kg)	71.4 ± 12.2
	BMI (kg/m^2^)	25.5 ± 3.4
	Waist circumference (cm)	87.0 ± 10.4
	Hip circumference (cm)	102.1 ± 9.5
	Waist to hip ratio	0.9 ± 0.1

**Table 4:** Cardiovascular, physiological, functional, metabolic and dietary characteristics of the participants included

Characteristics		Total *n* = 101 (presented as means ± SD)
BP (mmHg)	Systolic	146 ± 22
	Diastolic	86 ± 11
Resting HR (bpm)		65 ± 11
Blood biomarkers	Glucose (mg/dL)	101.5 ± 13.2
	HDL (mg/dL)	54.8 ± 12.8
	Triglycerides (mg/dL)	109.2 ± 41.0
	LDL (mg/dL)	137.9 ± 39.4
	Total cholesterol (mg/dL)	214.6 ± 4.3
6MWT	Total distance walked (m)	530.8 ± 79.7
	Walking speed (m/min)	88.5 ± 13.3
	RPE	11 ± 2
Estimated VO2max (ml/kg/min)		28.47 ± 6.9
Physical Activity	Sedentary time (min)	4927.3 ± 1254.1
	Light PA (min), (%)	8347 ± 1558.1
	Moderate PA (min), (%)	436.7 ± 252.4
	Vigorous PA (min), (%)	21.3 ± 45.1
	Very vigorous (min), (%)	2.8 ± 45.1
	Total MVPA (min)	500.3 ± 320.9
	Average steps/day	7418.2 ± 3201.2
Diet	Carb (g, % of energy)	184.5 ± 62.9 (45.7%)
	Protein (g, % of energy)	71.7 ± 20.5 (18.1%)
	Fat (g, % of energy)	63.9 ± 22.8 (35.5%)

*BMI* body mass index, *BP* blood pressure, *HR* heart rate, *6MWT* 6-min waking test, *RPE* Rate of perceived exertion. The percentages in the diet section represent the macronutrients intake as a percentage of total energy intake

### Overall gut microbiota composition

**Fig. 1:**
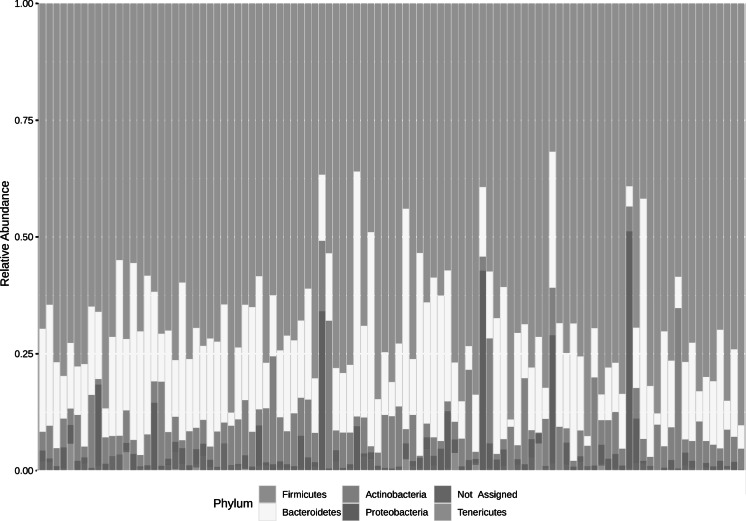
Distribution of phyla in the participants’ faecal microbiota. Each vertical bar corresponds to a participant

**Fig. 2:**
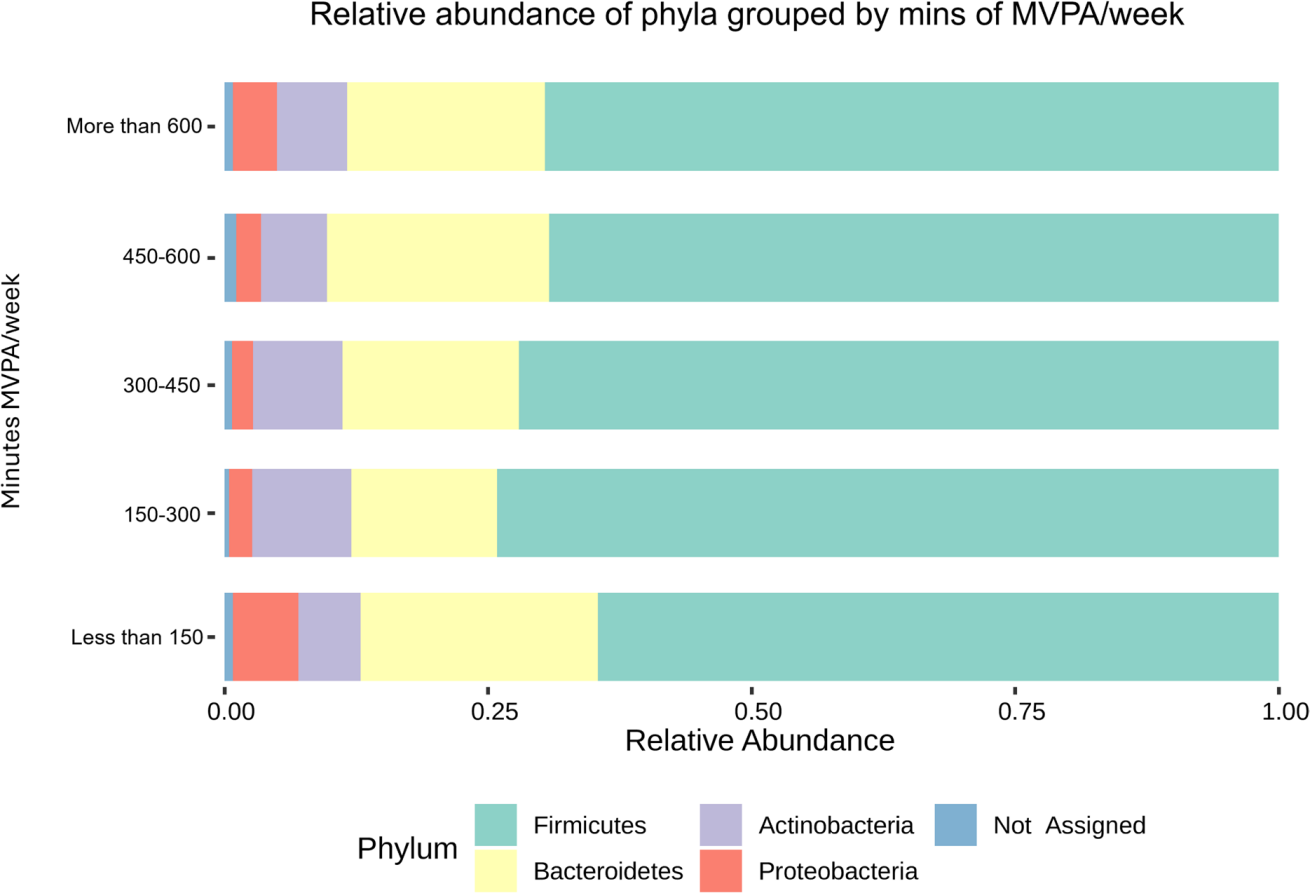
Relative abundance of phyla grouped by minutes of MVPA/week

Overall, 70% of the identified taxa belonged to Firmicutes, 18% to Bacteroidetes, 7% to Actinobacteria, 3% to Proteobacteria and less than 1% to Tenericutes. The relative abundance per sample of these phyla are presented in Fig. 1. When grouped by minutes of MVPA per week, those who were inactive or doing less than 150 min of MVPA per week had a higher abundance of Proteobacteria (6.2%) and lower abundance of Actinobacteria (5.9%) also when compared to all other groups (see Fig. 2).

### Alpha and beta diversity

**Fig. 3:**
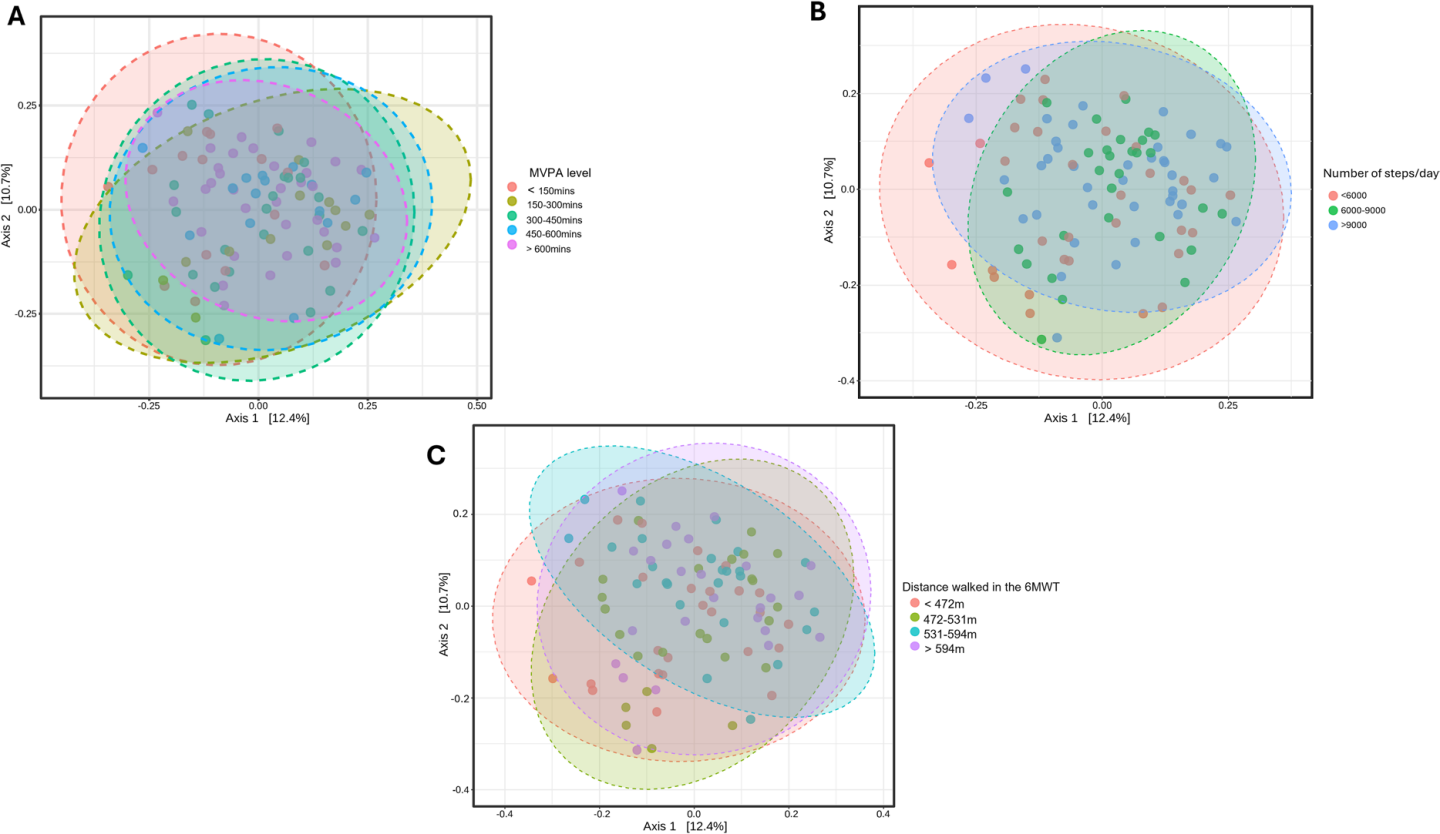
PCoA plots of Beta diversity based on Bray–Curtis dissimilarity measure. A- MVPA level; B- Number of steps per day; C- Distance walked in the 6MWT

There were no significant differences in alpha or beta diversity measures among individuals with varying physical activity levels, daily steps, or distances walked in the 6MWT (Fig. 3). The PCoA plots in Fig. 3 illustrate that the microbial composition does not vary substantially between the groups. However, this does not mean that PA does not affect the GM, it means that most likely there are other confounding factors that might be influencing the GM. Several differences in specific taxa were found in the differential abundance analysis.

### Differential abundance analysis

#### Minutes of MVPA/week

**Fig. 4:**
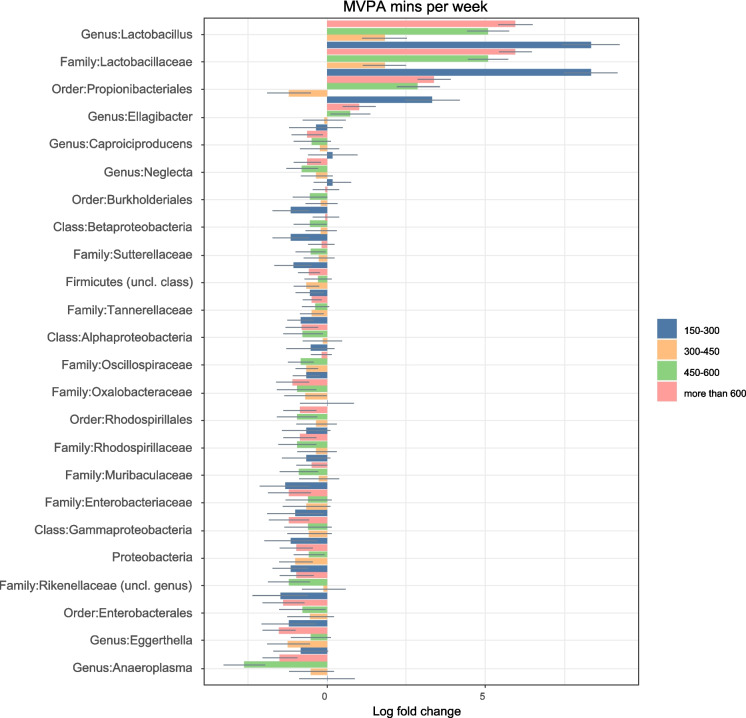

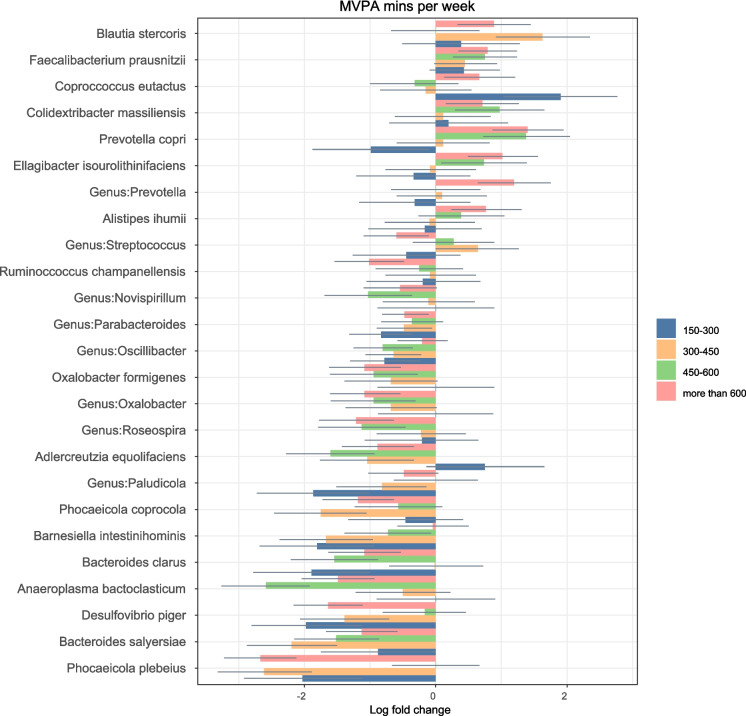
**a** Relative log-fold changes in the GM composition at various taxonomic levels between the different PA levels. The inactive group (less than 150 min of MVPA/week) was considered as the reference group. Therefore, all the log fold changes are relative to that group. Positive values denote taxa with higher relative abundances while negative values denote taxa with lower relative abundance. Only taxa that were statistically significant (FDR-adjusted p-value ≤ 0.05) are shown. The coloured bars represent the log-fold change while the grey lines indicate the standard error. uncl: unclassified. **b** Relative log-fold changes in the GM composition at various taxonomic levels between the different PA levels. The inactive group (less than 150 min of MVPA/week) was considered as the reference group. Therefore, all the log fold changes are relative to that group. Positive values denote taxa with higher relative abundances while negative values denote taxa with lower relative abundance. Only taxa that were statistically significant (FDR-adjusted p-value ≤ 0.05) are shown. The coloured bars represent the log-fold change while the grey lines indicate the standard error. uncl: unclassified

All of the results in this section (Fig. 4a and b) are compared to the participants who were inactive (less than 150 min MVPA/week) and the groups of MVPA were defined as multiples of recommended PA/week: 150–300 (between the recommended and 2 times the recommended), 300–450 (between 2 and 3 times the recommendation), 450–600 (between 3 to 4 times), and above 600 (above 4 times recommended PA). Additionally, they were all adjusted for diet and age, and all the calculations presented are significant after FDR correction.

The log fold changes for all taxa are presented in the supplementary file.

#### Taxa that were less abundant in those who were active compared to their inactive counterparts

A general decrease in the abundance of taxa belonging to the Proteobacteria phylum was observed in those older adults who were active. This included the Alpha, Gamma and Betaproteobacteria classes, the orders Burkholderiales, Enterobacterales and Rhodospirillales, the families Enterobacteriacae, Rhodospirillaceae, Sutterellaceae and Oxalobacter, the genera *Novispirillum, Oxalobacter* and *Roseospira*, and the *Desulfovibrio* and *Oxalobacter formigenes* species.

Furthermore, the families Anaeroplasmataceae, Muribaculaceae, Oscillospiraceae and Tannerellaceae and the genera *Eggerthella*, *Neglecta, Paludicola, Anaeroplasma, Caproiciproducens, Parabacteroides* and *Oscillospira* were also found to be in lower abundance in those who fulfilled the PA recommendations.

The species *Anaeroplasma bactoclasticum, Bacteroides clarus, Bacterois salyersiae*, *Phocaeicola plebeius, Phocaeicola coprocola*, *Ruminococcus champanellensis*, *Adlercreutzia equolifaciens, Ellagibacter isourolithinifaciens* (only decreased on the 150–300 and 300–400 min groups) and *Barnesiella intestinihominis* were also less abundant in active older adults.

#### Taxa that were more abundant in those who were active compared to their inactive counterparts

A large increase in the abundance of both the Lactobacillaceae family and *Lactobacillus* genus was observed in the faeces of active older adults. The *Lactobacillus* genus was more abundant in those who were active compared to those who were not.

In addition, there was a higher abundance of *Faecalibacterium prausnitzii* in the older adults who fulfilled the PA recommendations when compared to those who did not. The abundance of *F. prausnitzii* seemed to increase as the MVPA/week increased, suggesting a possible dose–response relationship between this species and the amount of weekly MVPA in older adults.

A greater abundance of Propionibacterales (except in the 300–400 min group) was observed in those who were active compared to inactive.

Focusing on genera, *Streptococcus* had a variable response to MVPA, since it was higher in the 300–450 min and 450–600 min groups and lower in the 150–300 min and above 600 min groups. *Ellagibacter* and its related species, *Ellagibacter isourolithinifaciens,* also had variable relationships with PA as they were less abundant in those who did between 150–450 min MVPA/week and more abundant in those who did above 450 min MVPA/week.

With regards to species, there was an increase in the abundance of *Blautia stercoris, Coproccocus eutactus, Alistipes ihumii, Colidextribacter massiliensis* and *Prevotella copri* (the latter had lower abundance in those with 150–300 min MVPA/week but then increased abundance as mins MVPA/week increased).

### Steps/day

**Fig. 5:**
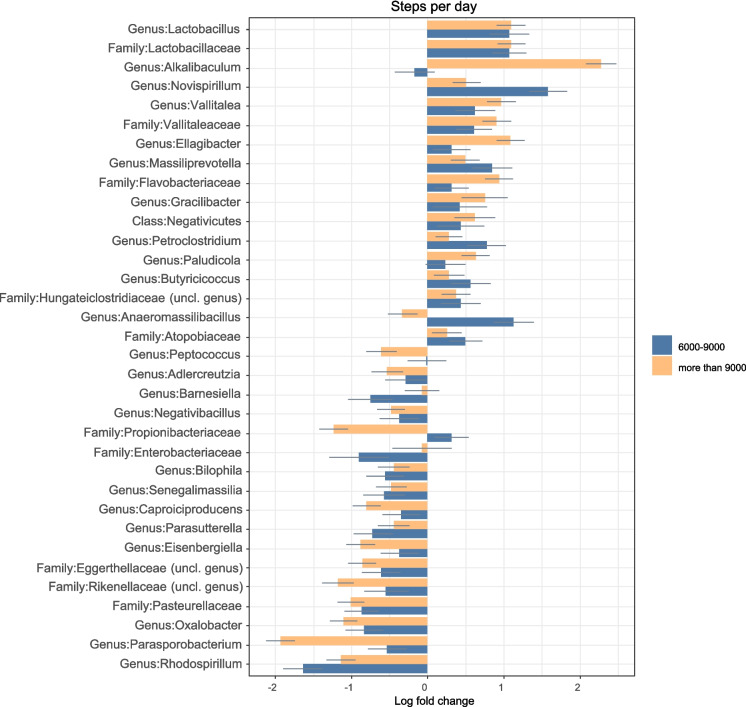

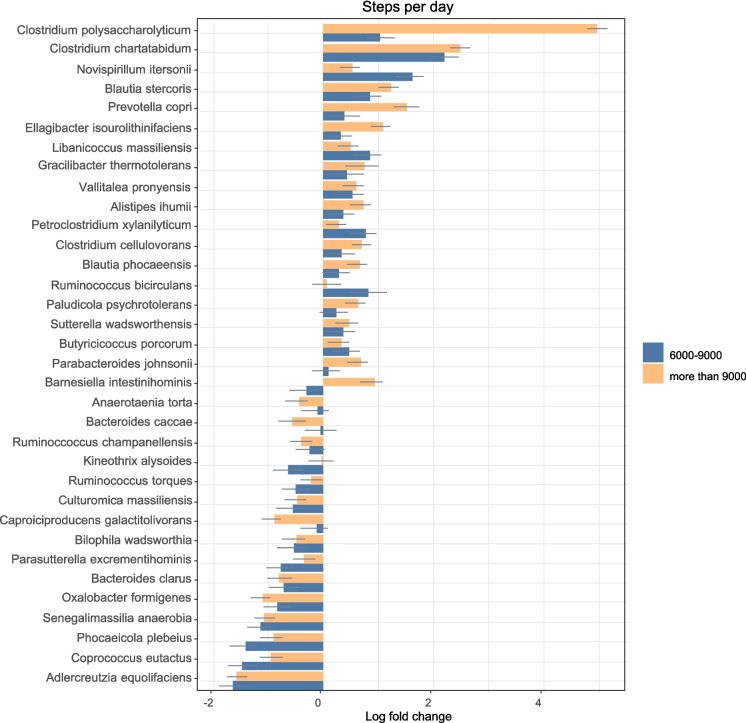
**a** Comparison of relative log-fold changes between participants who did less than 6000 steps/day (reference group) and those who did between 6000–9000 and more than 9000 steps/day. Positive values denote taxa with higher relative abundances while negative values denote taxa with lower relative abundance. Only taxa that were statistically significant (FDR-adjusted p-value ≤ 0.05) are shown. The coloured bars represent the log-fold change while the grey lines indicate the standard error. uncl: unclassified. **b** Comparison of relative log-fold changes between participants who did less than 6000 steps/day (reference group) and those who did between 6000–9000 and more than 9000 steps/day. Positive values denote taxa with higher relative abundances while negative values denote taxa with lower relative abundance. Only taxa that were statistically significant (FDR-adjusted p-value ≤ 0.05) are shown. The coloured bars represent the log-fold change while the grey lines indicate the standard error. uncl: unclassified

The groups in this section (Fig. 5a and b) were divided based on their step counts per day. Three groups were created: those who did less than 6000 steps/day, those who did between 6000–9000 steps/day and those who did more than 9000 steps/day. The 6000 steps/day cut-off was chosen due to a recently published meta-analysis that showed that there was a decreased risk of mortality in older adults who did above 6000 steps/day [61]. All the results were adjusted for diet and age, and all of the calculations presented are significant after FDR correction.

 The log fold changes for all taxa are presented in the supplementary file.

#### Taxa that were less abundant in those who did more steps per day

With regards to order, Pasteurellales (only in the above 6000 steps group) and Propionibacteriales (only in the above 9000 steps group) were less abundant.

Interestingly, several families from the Proteobacteria phylum (such as Enterobacteriaceae, Oxalobacteriaceae, Pasteurellaceae) along with Eggerthellaecae and Rikenellaceae were less abundant in the older adults who did more than 6000 steps/day.

There were also several genera that had lower relative abundance in those who did more than 6000 steps/day when compared to those who did not such as *Adlercreutzia, Barnesiella, Bilophila, Caproiciproducens, Eisenbergiella, Negativibacillus, Oxalobacter, Parasporobacterium, Parasutterella, Peptococcus, Rhodospirillum* and *Senegalimassilia*.

The species *Ruminococcus torques, Kineothrix alysoides, Senegalimassilia anaerobia, Bacteroides caccae, Ruminococcus champanellensi, Bacteroides clarus, Adlercreutzia equolifaciens, Coprococcus eutactus, Parasutterella excrementihomonis, Oxalobacter formigenes, Caproiciproducens galactitolivorans, Culturomica massiliensis, Anaerotaenia torta* and *Bilophila wadsworthia* were all less abundant in the gut microbiota of older adults who did more than 6000 steps/day.

#### Taxa that were more abundant in those who did more steps per day

Negativicutes and Flavobacteriia classes were more abundant in those who did more steps/day. With regards to families, Flavobacteriaceae, Atopobiaceae, Gracilibacteraceae, Vallitaleaceae and Hungateiclostridiaceae were all more abundant in those who did more than 6000 steps/day.

Just like when participants were grouped according to MVPA/week, Lactobacillaceae and *Lactobacillus* were also more abundant in older adults who did more than 6000 steps/day.

The genera *Alkalibaculum, Butyricicoccus, Ellagibacter, Gracilibacter, Massiliprevotella, Petroclostridium, Vallitalea* and the species *Clostridium polysaccharolyticum, Ruminococcus bicirculans, Clostridium cellulovorans, Clostridium chartatibidum, Prevotella copri, Alistipes ihumii, Barnesiella intestinohominis, Ellagibacter isourolithinifaciens, Novispirillum itersonii, Parabacteroides johnsonii, Libanicoccus massiliensis, Balutia phocaeensis, Butyricoccus porcorum, Vallitalea pronyensis, Paludicola psychrotolerans, Blautia stercoris, Gracilibacter thermotolerans, Sutterella wadswothensis* and *Petroclostriidum xylanilyticum* were all more abundant in those who did above 6000 steps/day. Interestingly, there was a very high abundance of some *Clostridium* species such as of *C. polysaccharolyticum* and *C. chartatabidu*m in those who did more steps/day.

### Distance walked in the 6MWT

**Fig. 6:**
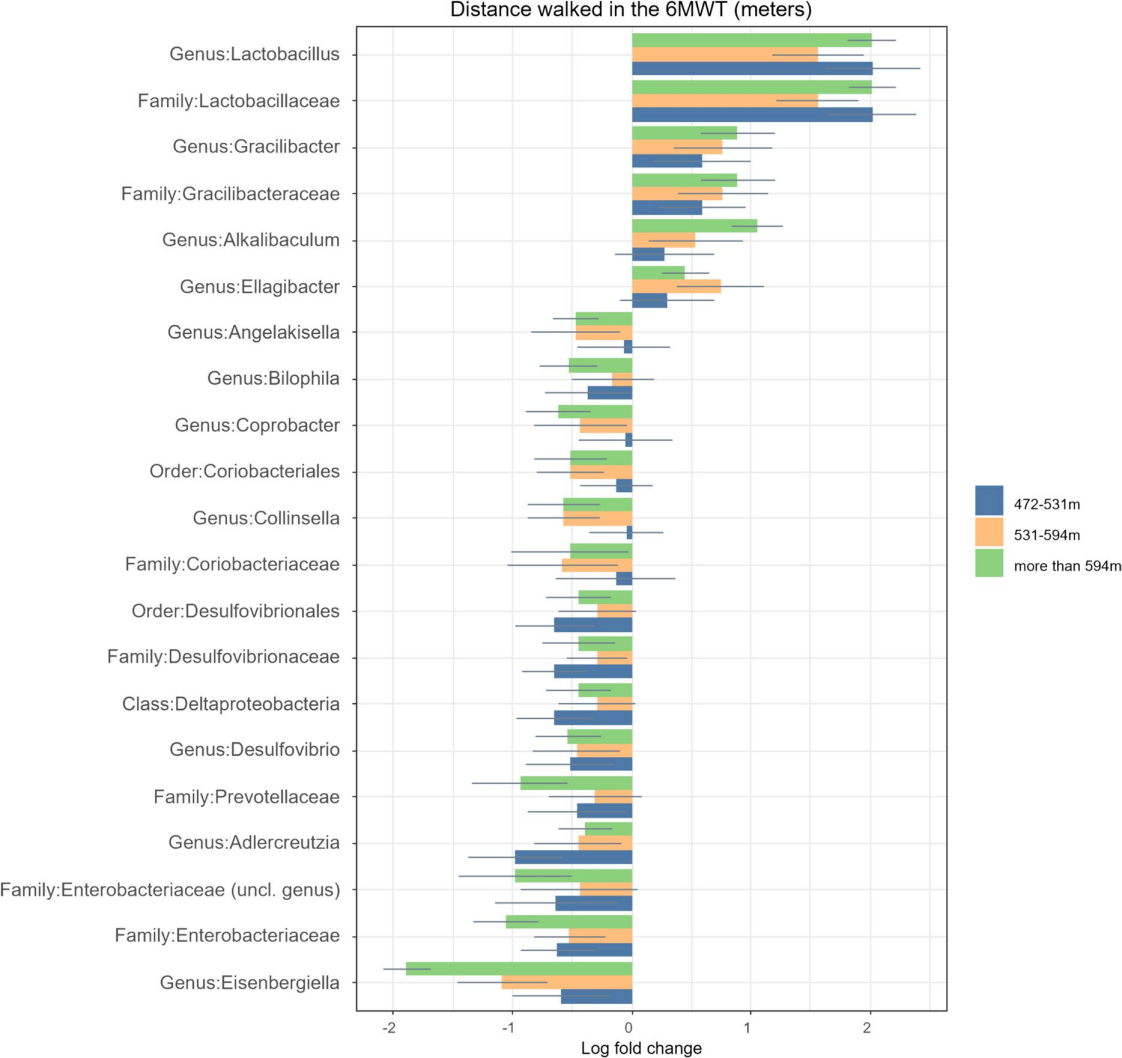

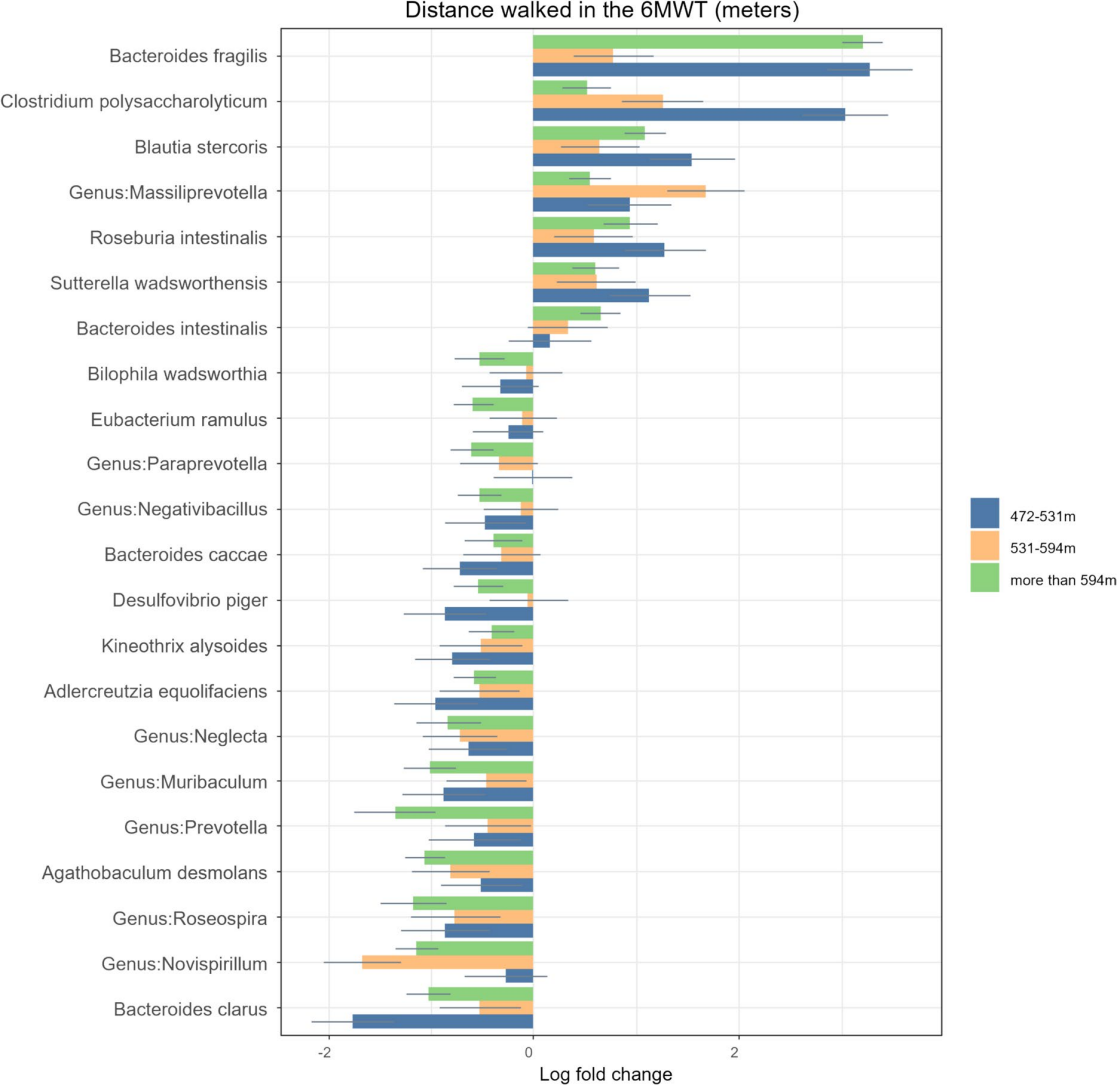
**a** Comparison of relative log-fold changes between participants who walked less than 472 m in the 6MWT (reference group) and those who walked between 472–531, 531–594 and more than 594 m. Positive values denote taxa with higher relative abundances while negative values denote taxa with lower relative abundance. Only taxa that were statistically significant (FDR-adjusted p-value ≤ 0.05) are shown. The coloured bars represent the log-fold change while the grey lines indicate the standard error. uncl: unclassified. **b** Comparison of relative log-fold changes between participants who walked less than 472 m in the 6MWT (reference group) and those who walked between 472–531, 531–594 and more than 594 m. Positive values denote taxa with higher relative abundances while negative values denote taxa with lower relative abundance. Only taxa that were statistically significant (FDR-adjusted p-value ≤ 0.05) are shown. The coloured bars represent the log-fold change while the grey lines indicate the standard error. uncl: unclassified

The groups in this section (Fig. 6a and b) were divided into quartiles according to: those who walked less than 472 m, those who walked between 472–531 m, those who walked between 531–594 m and those who walked more than 594 m in the 6MWT. The distance walked was used in the 6MWT as a proxy for cardiorespiratory fitness and functionality. All results were adjusted for diet and age, and all of the calculations presented are significant after FDR correction.

 The log fold changes for all taxa are presented in the supplementary file.

#### Taxa that were less abundant in those who walked further in the 6MWT

The Deltaproteobacteria class, Desulfovibrionales order and the Desulfovibrionaceae, Enterobacteriaceae and Coriobacteriacae families were all less abundant in the older adults who walked further than 472 m during the 6MWT.

Several genera such as *Angelakisella, Bilophila, Collinsella, Desulfovibrio, Eisenbergiella, Muribaculum, Negativibacillus, Neglecta, Novispirillum, Paraprevotella, Prevotella, Roseospira* and *several species such as Kineothrix alysoides, Bacteroides caccae, Bacteroides clarus, Agathobaculum desmolans, adlercreutzia equolifaciens, Desulfovibrio piger, Eubacterium ramulus* and *Bilophila wadsworthia* were also less abundant in those who walked further than 472 m in the 6MWT.

#### Taxa that were more abundant in those who walked further in the 6MWT

Lactobacillaceae and *Lactobacillus* were of higher abundance in those who walked more than 472 m during the 6MWT. Besides Lactobacillaceae, other families which were also more abundant included Gracilibacteriacae and Prevotellaceae. The genera *Alkalibaculum, Ellagibacter, Gracilibacter, Massiliprevotella* and the species *Bacteroides fragilis, Roseburia intestinalis, Bacteroides intestinalis* and *Blautia stercoris* were also more abundant in those who walked more than 472 m during the 6MWT.

## Discussion

The aim of this study was to compare the faecal microbiota composition of community dwelling older adults with different PA levels, amounts of steps/day, and their performance on the 6MWT as a proxy for cardiorespiratory fitness. Overall, our findings show that active older adults, those who do more steps/day, and walked further in the 6MWT, had higher abundance of health-related bacteria and lower abundance of pathogenic-related bacteria when compared to their inactive counterparts. More specifically, those who fulfilled PA recommendations had higher abundances of health-related taxa such as *Lactobacillus* and *F. prausnitzii* and a decrease in the abundance of pathogens and taxa associated with disease such as Enterobacteriacae, Sutterellaceae and *D. piger*. Additionally, those who did more steps per day also had a higher abundance of health-related bacteria such as *Lactobacillus*, and butyrate-producing species belonging to *Clostridium* cluster XIVa and reduced abundance of pathobionts such as taxa belonging to the Proteobacteria phylum (including *B. wadsworthia*). Finally, with regards to distance walked in the 6MWT, the results suggest that there might be a connection between the gut microbiota and cardiorespiratory fitness, functional capacity and gait speed. According to our results, older adults who walk further in the 6MWT (and therefore have higher functional capacity and faster gait speed) have higher abundance of health-related bacteria such as *Lactobacillus, Roseburia intestinalis and Blautia stercoris,* and lower abundance of disease-associated taxa such as *Desulfovibrio* and *Bilophila.*

These results suggest that not only PA, but also steps/day and performance on the 6MWT (cardiorespiratory fitness and functionality) are associated with differences in the composition of the gut microbiota of older adults through an increase in health-related taxa and a decrease in the abundance of pathogenic taxa. These compositional changes suggest improvements in the lower gut microbiota composition of older adults which may be central to reversing age-associated dysbiosis and promoting healthy aging.

One of the most striking findings of this study was the high abundance of the Lactobaccillaceae family and *Lactobacillus* genus in active older adults when compared to the inactive group. For example, older adults who did between 150–300 min of MVPA/week had 8 log-fold change (lfc) higher abundance of this genus when compared to those who did not reach the PA recommendations. Additionally, in those who did more steps/day and walked further in the 6MWT, abundance of this family and genus was also higher. These findings align with those from previous studies. A pilot study [43] showed a large increase in Lactobacillaceae after a 24-week combined exercise intervention in people aged 50–75 years old. Additionally, Lactobacillaceae was found to be significantly reduced by 26-fold in older adults with high frailty scores [62]. Several species belonging to this genus are well known probiotics and have been shown to have health-related properties. The mechanisms behind the beneficial effect of lactobacilli may be because they are the main producers of lactic acid in the intestine which gives them the ability to alter the luminal pH and inhibit the growth of Gram-negative bacteria species such as *E.coli* or *Salmonella enterica* [63]. Additionally, they have also been reported to regulate the immune system through inhibition of pro-inflammatory cytokines IL-12 and IL-8 and by stimulating the production of anti-inflammatory IL-10 [64].

Another interesting finding was the higher abundance of *Faecalibacterium praunsnitzii* in those who were active compared to those who were not. Intriguingly, this species’ abundance seems to increase as PA levels increase, suggesting that there may be a dose response relationship between it and PA levels. However, more studies are needed to properly ascertain this relationship particularly as *F. praunsnitzii* has been shown to be reduced in centenarians [65], and to be negatively associated with aging [66]. In a previous study comparing the gut microbiota of senior orienteering athletes and community dwelling older adults, *F. prausnitzii’s* abundance was significantly different between those two groups with the senior orienteering athletes having higher abundance [44]. Additionally, in a cross-sectional study involving women aged between 18–40 years, this species was also more abundant in those who were active [39]. Taken together, these findings suggest that exercise or PA can increase the abundance of this beneficial microbe, thereby counteracting the aging-associated changes in its abundance. *F. prausnitzii* is one of the most copious producers of butyrate in the gut. It converts acetate to butyrate and also synthesizes other anti-inflammatory molecules such as shikimic and salicylic acids [67]. These molecules act together to block NF-kB activation, IL-8 production and TNF-α secretion thereby promoting an anti-inflammatory environment [68].

Another finding of note was the very high abundance of *Clostridium polysaccharolyticum* in the lower gut microbiota of older adults who did more than 9000 steps/day. *C.polysaccharolyticum* belongs to the *Clostridium* cluster XIVa which consists of several species known to be able to ferment several nutrients and produce acetate, propionate and butyrate [69]. Besides producing SCFAs, species from this cluster can also promote the accumulation of Treg cells in the colon and block NF-kB and IL-8 production [70], thereby promoting an anti-inflammatory gut environment. Moreover, they might be able to promote gut homeostasis by controlling the number of resident gut pathogens [71]. Additionally, two other members of the *Clostridium* genus, both butyrate producers [72, 73] – *Clostridium cellulovorans* and *Clostridium chartatabidum* – were found to be enriched in the lower guts of those who did more steps/day.

In this study, we also observed a general reduced abundance of members of the Proteobacteria phylum in participants who were active compared to those who were not. Bacteria belonging to the Proteobacteria phylum and Alpha, Beta and Gammaproteobacteria classes were less abundant in active older adults. Additionally, members of the Enterobacteriaceae, Sutterellaceae and Rhodospirillaceae families were also less abundant in active older adults when compared to the inactive ones, and those who did more steps/day and walked further in the 6MWT also had lower abundance of Enterobacteriaceae. These findings agree with previous studies that demonstrated that an exercise intervention reduced the abundances of Proteobacteria and Enterobacteriaceae in obese sedentary women [74] and the abundance of Proteobacteria in obese children [75]. This phylum, more specifically Enterobacteriaceae, have been strongly associated with gastrointestinal causes of death [76] and with inflammation since they are involved in the production of several endotoxins such as lipopolysaccharide (LPS), trimethylamine-N-oxide (TMAO) which then activate the inflammation cascades of the immune system [77]. There are several species of pathogenic bacteria that belong to this group. Interestingly, Enterobacteriaceae abundance was found to be seven-fold higher in very frail older adults [62].

*Bilophila* and *Bilophila wadsworthia* were less abundant in those who did more steps/day and in those who walked further in the 6MWT. This is potentially a positive effect of increased steps/day as the transfer of *B. wadsworthia* from human to germ-free mice caused systemic inflammation and an increase in serum amyloid A (SAA) and IL-6, which are both pro-inflammatory cytokines [78]. In addition to its pro-inflammatory effects, *B. wadsworthia* also promotes intestinal barrier permeability, dysregulation of bile acid metabolism and changes in the gut microbiome functional profile [79].

Also, within the Proteobacteria phylum, *Desulfovibrio piger* – often associated with negative health effects – was less abundant in a) those who fulfilled the PA recommendations compared to those who did not and b) those who walked further in the 6MWT. *D. piger* is a sulphate-reducing bacteria that generates hydrogen sulphide, which is a toxic substance that can damage the intestinal barrier, inhibit fatty acid oxidation and cause neurodegenerative diseases through changes in mitochondrial respiration by causing DNA damage [80, 81]. This species was previously reported to be enriched in patients with sarcopenia, was positively associated with sarcopenia severity [81] and was significantly higher in those with inflammatory bowel diseases [82].

The fact that these genera and species were reduced in those who did more steps/day and walked further in the 6MWT suggest, yet again, a protective beneficial role of being physically active in older age.

Less well studied, but still potentially interesting, the abundance of *Ruminococcus champanellensis* was lower in those who were active and in those who did more steps/day. *R. champanellensis* has previously been found to be present in higher proportions in western diet-fed animals where it was positively correlated with body fat and blood cortisol concentration [83].

Our study found that the abundance of many bacteria was positively associated with PA levels/number of steps per day/6MWT distance. *Prevotella copri* was more abundant in the active group and in those who did more steps/day. *P. copri* is the most common *Prevotella* species found in the human gut and there are contrasting results in the literature regarding its impact on host health. Some studies have shown that it is associated with diseases [84, 85] while others show that they are associated with health and fibre consumption [86, 87]. These contrasting findings are because this species contains several strains that differ widely in their effects on host health [88]. Since most gut microbiome studies only investigate up to species level, it is difficult to establish a robust association with health and/or disease and the different strains. More studies are therefore needed to determine how different *P. copri* strains relate to health and/or disease outcomes.

*Blautia stercoris*, a less studied species that has previously been shown to attenuate social deficits and anxiety in an autism mouse model [89] and to be significantly decreased in children with Downs syndrome [90], was more abundant in the active group and in those who did more steps/day and walked further in the 6MWT. This suggests that this species might be beneficial for health.

*Roseburia intestinalis* was more abundant in those who walked longer distances in the 6MWT. *R. intestinalis* is one of the most abundant butyrate-producers in the gut [91] and it has been positively associated with health in that it can promote an anti-inflammatory environment, promote gut barrier integrity, the regulation of the differentiation of regulatory T-cells and, suppression of Th17 and macrophages through its flagellin and butyrate production [92]. Moreover, this species’ abundance was found to be lower in individuals with low bone mineral density [93] and was negatively correlated with waist circumference [94]. Furthermore, the administration of *R. intestinalis* alleviated symptoms of ulcerative colitis [95] and was able to suppress Crohn’s disease pathogenesis [96, 97] through its anti-inflammatory properties.

Some species from the *Bacteroides* genus were less abundant in those who were more active, did more steps/day or walked further in the 6MWT. For example, *Bacteroides clarus*, a species that has been previously associated with cancer [98], diabetes type 2, acute myocardial infarction [99] and inflammation [100] was less abundant in those who were active, in those who did more steps, and in those who walked further in the 6MWT. Additionally, *Bacteroides caccae*, a species that was enriched in subjects with diabetes type 2 [101] and in subjects with an inflammatory diet [100], was also less abundant in those who walked a further distance in the 6MWT and did more steps/day. *Bacteroides salyersiae’s* abundance was lower in active older adults compared to the inactive ones. Interestingly, in previous literature, this species was enriched in those consuming high fibre diets [102] and decreased in elderly patients with mild cognitive impairment [103] suggesting that it might be beneficial to health. Since 16 s rRNA sequencing only provides resolution up to species level, there might be different strains with different functions and further research is needed to elucidate the role of *B. salyersiae* and its strains on health and disease.

Another interesting result in our study was the higher abundance of *Bacteroides fragilis* in those who walked further distance in the 6MWT. Literature still does not agree with regards to the role of *B. fragilis* in health and/or disease and infection, with some studies showing that it is a pathogenic bacteria [104] and others showing that it is an health-related taxa and should be considered as a next generation probiotic [105]. Again, its benign or pathogenic role might be due to different strains [106] and since most of the work that has been done in gut microbiota studies in health or disease has employed 16 s rRNA sequencing, we cannot know which strain was present, therefore more studies are needed to further elucidate the roles of the specific strains in health and disease.

In our study *Adlercreutzia equolifaciens’* abundance was reduced in those who were more active, in those who did more steps/day and in those who walked further in the 6MWT. A previous study that employed a 12-week HIIT exercise intervention in older adults with celiac disease [107] also found that the genus *Adlercreutzia* was more abundant in the non-exercise group when compared to the exercise group. Additionally, this species was also associated with back pain and with BMI [108, 109]. In contrast, an observational study performed by [45] reported that older men who did more steps/day had higher abundances of *Adlercretuzia*, and a study conducted by [110] found that this genus is anti-inflammatory and that it might have therapeutic potential to improve liver diseases. These contrasting results might be due to either different strains of the same species, different techniques used and/or different variable regions analysed or even due to population differences. More research is needed to properly ascertain the role of this species and its strains on the health of older adults.

Figure 2 shows that individuals who were inactive had a higher relative abundance of Proteobacteria and a lower abundance of Actinobacteria. Conversely, the other MVPA groups exhibited the opposite trend, except for those engaging in more than 600 MVPA minutes per week, who showed a similar trend to the inactive group regarding these two phyla. This pattern may be explained by the relationship between exercise intensity, duration, and gut stress. While MVPA generally has beneficial effects on the GM, longer durations of MVPA might induce physiological stress in the gut [111, 112], altering the gut environment and shifting the GM composition towards a more inflammatory state (e.g., increased Proteobacteria and decreased Actinobacteria). However, future studies are needed to further elucidate this result.

The strengths of the study are: 1) the objective measurement of PA and steps (using accelerometers) for a 7-day period; 2) the FFQ questionnaire which provided a very complete description of participants’ diet, components of which were used as covariates in the analysis; and 3) the inclusion and exclusion criteria which made sure no participants with certain diseases/conditions or taking certain types of medication that directly affected the gut microbiome were enrolled, providing a clearer picture of the effects of PA on the gut microbiota of older adults.

The weaknesses of the study were: 1) the cross-sectional design, meaning that causation cannot be established; 2) the use of 16 s rRNA next generation sequencing. Despite it being one of the most widely used techniques to analyse gut microbiota, it has its disadvantages such as low resolution, inability to provide functional profiling and the inherent bias related to the selection of primers and targeted hypervariable regions; 3) the sample population was more active and fitter than average; 4) use of faecal samples to assess lower gut microbiota and 5) some of the participants in this study engaged in cycling activities and the accelerometer placed on the waist might have not recorded this activity accurately.

Observed differences in the GM composition of older adults who engaged in different levels of PA highlight important practical implications for promoting healthy ageing. Our findings suggest that encouraging older adults to meet the WHO/UK PA recommendations may not only improve physical and overall health but also improve their GM composition, potentially reversing age-related dysbiosis. These results could inform the development of future interventions to promote healthy ageing and to use exercise/physical activity as a non-pharmacological tool to improve health in older age. While our findings provide insights into differences of the GM composition of older adults with different PA levels, and it shows that those adults who meet the WHO recommendations have a healthier GM profile, it is important to acknowledge the cross-sectional nature of this study and this ldoes not allow the establishment of causality. Future research should focus on performing well controlled longitudinal studies to assess the effects of PA on the GM of older adults and its mechanistic implications for healthy ageing.

## Conclusion

In conclusion, this study showed that community dwelling older adults who are active and do at least 150 min of MVPA/week, those who do more than 6000 steps per day, and those who performed better in the 6MWT (walked further distance, have higher gait speed and therefore higher functionality) had a higher abundance of health-related bacteria and lower abundance of well-known gut pathobionts when compared to those who did not reach the PA recommendations, those who did less than 6000 steps/day, or those who walked less than 472 m in the 6MWT. These findings emphasize the importance of PA in older adults and highlight a connection between the gut microbiota, PA and healthy aging, suggesting that PA might be used as a tool to support gut health and consequently promote healthy aging. Future studies should focus on the effects of endurance and/or resistance exercise interventions on the gut microbiota of older adults. Additionally, gut-derived metabolomics analysis should be used in order to understand the functional profile of the gut microbiota and its relationship with PA (going from ‘who is there’ to ‘what are they doing there’) to better understand this connection with the gut microbiota, healthy aging and PA.

## Supplementary Information

Below is the link to the electronic supplementary material.

Supplementary file1 (XLSX 20 KB)

Supplementary file2 (XLSX 20 KB)

## Data Availability

The data that supports the findings of this study are available on request from the corresponding author, CR. The data are not publicly available due to containing information that could compromise the privacy of research participants. The data is pseudonymised and has been uploaded to Zenodo under the DOI: 10.5281/zenodo.12799150 and its access is restricted.
